# Development and external validation of a novel nomogram model for predicting postoperative recurrence-free survival in non-muscle-invasive bladder cancer

**DOI:** 10.3389/fimmu.2022.1070043

**Published:** 2022-11-15

**Authors:** Li Ding, Xiaobin Deng, Wentao Xia, Kun Wang, Yang Zhang, Yan Zhang, Xianfeng Shao, Junqi Wang

**Affiliations:** ^1^ Department of Urology, the Affiliated Hospital of Xuzhou Medical University, Xuzhou, Jiangsu, China; ^2^ Department of Urology, the First Affiliated Hospital of Guangxi Medical University, Nanning, Guangxi, China

**Keywords:** predictive indicator, risk factor, bladder cancer, NMIBC, tumor recurrence, risk calculator, nomogram

## Abstract

**Background:**

Transurethral resection of the bladder tumor with or without adjuvant intravesical instillation (IVI) has been the standard treatment for non-muscle-invasive bladder cancer (NMIBC), whereas a high percentage of patients still experience local tumor recurrence and disease progression after receiving the standard treatment modalities. Unfortunately, current relevant prediction models for determining the recurrent and progression risk of NMIBC patients are far from impeccable.

**Methods:**

Clinicopathological characteristics and follow-up information were retrospectively collected from two tertiary medical centers between October 2018 and June 2021. The least absolute shrinkage and selection operator (LASSO) and Cox regression analysis were used to screen potential risk factors affecting recurrence-free survival (RFS) of patients. A nomogram model was established, and the patients were risk-stratified based on the model scores. Both internal and external validation were performed by sampling the model with 1,000 bootstrap resamples.

**Results:**

The study included 299 patient data obtained from the Affiliated Hospital of Xuzhou Medical University and 117 patient data obtained from the First Affiliated Hospital of Guangxi Medical University. Univariate regression analysis suggested that urine red blood cell count and different tumor invasion locations might be potential predictors of RFS. LASSO-Cox regression confirmed that prior recurrence status, times of IVI, and systemic immune-inflammation index (SII) were independent factors for predicting RFS. The area under the curve for predicting 1-, 2-, and 3-year RFS was 0.835, 0.833, and 0.871, respectively. Based on the risk stratification, patients at high risk of recurrence and progression could be accurately identified. A user-friendly risk calculator based on the model is deposited at https://dl0710.shinyapps.io/nmibc_rfs/.

**Conclusion:**

Internal and external validation analyses showed that our model had excellent predictive discriminatory ability and stability. The risk calculator can be used for individualized assessment of survival risk in NMIBC patients and can assist in guiding clinical decision-making.

## Introduction

Bladder cancer is a common malignancy of the urinary system (1), which can be divided into two groups based on pathological stage: non-muscle-invasive bladder cancer (NMIBC) and muscle-invasive bladder cancer (MIBC) (2). MIBC accounts for approximately 30% of all cases, and even after radical cystectomy, the prognosis of patients in this group remains poor. On the other hand, NMIBC, which includes Ta, T1, and carcinoma *in situ* (CIS) stage, has a relatively good prognosis. The standard treatment for NMIBC has been transurethral resection of the bladder tumor (TURBT), with or without adjuvant intravesical instillation (IVI) of chemotherapy or bacillus Calmette-Guérin (BCG) therapy (3, 4). However, 40%-80% of initially treated NMIBC patients experience tumor recurrence, and approximately 15% eventually progress to MIBC (5–7). The risk-scoring models developed by the European Organisation for Research and Treatment of Cancer (EORTC) (3) and the Spanish Urological Organization (Club Urologico Español de Tratamiento Oncologico, CUETO) (4) have been widely used to predict the survival and prognosis of patients with NMIBC (8, 9). However, the above models did not take into account variables such as emerging biomarkers and tumor involvement sites.

The duration and intensity of tobacco smoking were already identified as the most important risk factors for the occurrence of bladder cancer and are considered to be also closely associated with its recurrence and progression (10–12). Interestingly, previous studies have suggested that differences in bladder tumor invasion locations may influence tumor pathology and patient prognosis (13–18). However, these studies have produced conflicting results and are limited by small sample sizes, variable study populations, and inconsistent definitions of tumor locations. Furthermore, systemic inflammatory response (SIR) indicators were also suggested to be associated with the prognosis of NMIBC (19–22). The fluctuations in the neutrophil-to-lymphocyte ratio (NLR) (23–26), platelet-to-lymphocyte ratio (PLR) (19, 22), systemic immune-inflammation index (SII) (27–30), and other SIR indicators have all been considered to have available predictive values for NMIBC patients’ survival. In addition, gross hematuria (31) and elevated body mass index (BMI) (32, 33) have also been described in previous studies as risk factors associated with NMIBC outcomes.

Previous studies have only focused on the analysis of newly identified individual risk factors. Due to the lack of a comprehensive evaluation, it was difficult to find the interrelationships between these variables, and therefore the performance of existing risk prediction models was limited. Meanwhile, models based on European and American cohorts are not necessarily applicable to Chinese populations. For the first time, in this study, we included urine red blood cell count (U-RBC) and times of IVI, along with other previously described statistically significant predictors, upon a full evaluation. Our study aimed to improve the predictive performance of survival outcomes for NMIBC patients and to demonstrate the feasibility and performance of the novel model in the real world through rigorous validation. Ultimately, we intended to establish a user-friendly risk calculator that is convenient for clinicians to utilize.

## Materials and methods

### Study population

**Figure 1:**
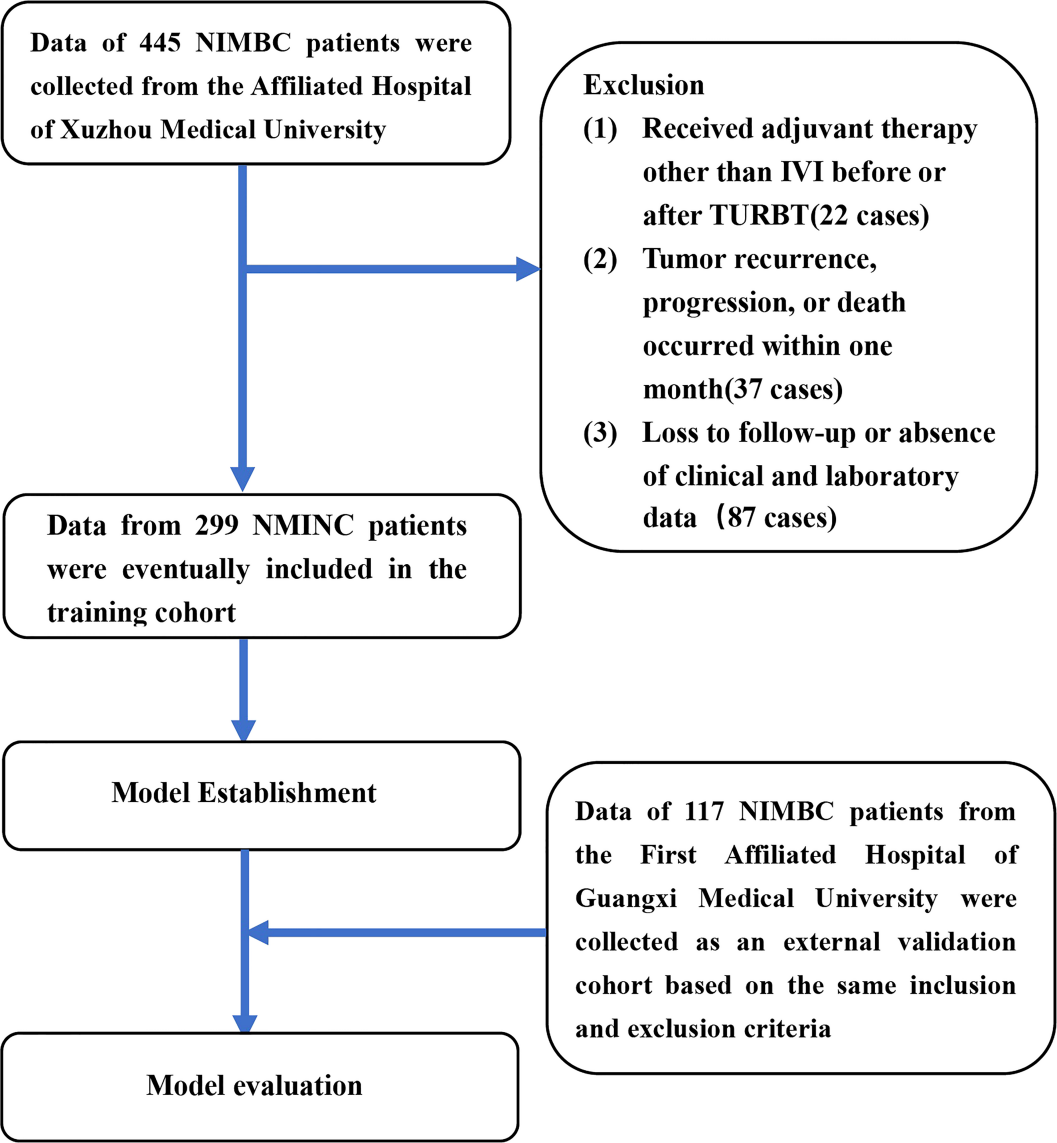
The flow chart for study inclusion and exclusion in the training cohort and external validation cohort. NMIBC, non-muscle-invasive bladder cancer; IVI, intravesical instillation; TURBT, transurethral resection of the bladder tumor.

This study complies with the Declaration of Helsinki and was approved by the Ethics Committee of the Affiliated Hospital of Xuzhou Medical University (XYFT2022-KL340-01) and the Ethics Committee of the First Affiliated Hospital of Guangxi Medical University (2022-E318-01). The requirement for written informed consent has been waived due to the retrospective study design. The medical records from the two tertiary medical centers were retrospectively searched for data on patients who were clinically diagnosed with “urothelial carcinoma of the bladder” from October 2018 to June 2021. The inclusion criteria used were as follows (1): patients with clear primary lesion (2); patients who had undergone a clear surgical approach (3); patients with postoperative histopathology confirming urothelial carcinoma of the bladder (4) patients with clear pathological staging records after surgery of non-muscle-invasive, and no lymph node invasion or distant metastasis (5); patients with complete clinical, laboratory, and follow-up data. Meanwhile, the exclusion criteria used were as follows (1): patients who received adjuvant therapy other than IVI before or after TURBT (2); patients with comorbid cancers (3); patients with tumor recurrence, progression, or death that occurred within one month from surgery. A study flow chart is shown in **Figure 1**.

Clinicopathological characteristics and follow-up information, including gender, age, BMI, smoking history, tumor status, previous symptoms, therapeutic schedule, etc., were collected. The pathological staging was evaluated according to the 2017 Tumor, Node, Metastasis classification of urinary bladder cancer (34). Meanwhile, the histological grading classification was performed following the World Health Organization 2004/2016 system. Preoperative inflammation-related markers, nutrition-related markers, and urine routine indices were also selected as potential predictive indicators. All laboratory parameters were collected within one week before surgery. Recurrence-free survival (RFS) was defined as the time from TURBT to the first evidence of either recurrence or progression, cancer-related death, or last follow-up.

### Adjuvant intravesical instillation therapy

The perfusion drugs used in the study include BCG, pirarubicin, gemcitabine, epirubicin, and hydroxycamptothecin. Except for a few patients who refused postoperative IVI, all other patients received their immediate single instillation (SI) within 24 hours after TURBT, and then once a week for six to eight weeks (induction perfusion). The frequency of IVI was then changed to once a month, with a recommended duration of twelve months (persistence perfusion).

### Statistical analysis

The indicators were transformed from continuous to categorical variables using the X-tile program[35], then compared using Chi-square tests. The Kaplan–Meier method was used to determine the clinical endpoints of patients, and the log-rank test was used to analyze them. The least absolute shrinkage and selection operator (LASSO) method was adopted for variable selection. The chosen variables were then included in the Cox regression analysis for calculating the hazard ratio (HR) with a 95% confidence interval (CI) to identify independent risk predictors. The nomogram was validated using Harrell’s concordance index (C-index). Meanwhile, the area under the receiver operating characteristics (ROC) curve (AUC) was used to evaluate the discrimination ability, and calibration curves were used to determine the calibration ability of the model. Both discrimination and calibration were assessed by bootstrapping with 1,000 resamples. The integrated discrimination improvement (IDI) (35) was used to show the improvement in the predictive accuracy of the nomogram. Decision curve analysis (DCA) (36) was performed to determine the clinical net benefit associated with using the predictive models at different threshold probabilities in the patient cohort. After building the model, the patients were stratified into low-risk, intermediate-risk, and high-risk groups by calculating the total points of individual patients and further evaluating the statistical differences between the groups. The programs X-tile 3.6.1 (http://tissuearray.org/), SPSS 26.0 (IBM Corp., Chicago, IL), and R 4.1.2 (http://www.R-project.org/) were used in performing the statistical analyses. A two-sided P-value<0.05 was considered to be statistically significant.

## Results

### Patient characteristics

In the training cohort, the data of 445 patients were collected, but after the screening, only 299 cases were finally included. The cut-off values for age at diagnosis and maximum tumor diameter were confirmed according to the latest European Association of Urology (EAU) criteria (37). The median follow-up time was 23 months (mean: 24 months, range: 1-45 months). The majority of patients were male (83.612%) and were younger than 70 years old (60.525%). Overweight subjects accounted for nearly half of the patients (46.154%). The majority of the tumors were classified in the Ta stage (60.535%), primary status (88.294%), high grade (63.211%), and less than 30 millimeter(mm) in size (73.913%). Nearly 60% of patients denied a smoking history. Most patients reported a history of painless gross hematuria in their previous self-reported symptoms or at follow-up (79.933%). Our medical center recommends that NMIBC patients receive SI followed by 6 to 8 weeks of induction perfusion and then 12 weeks of persistence perfusion after TURBT, and the training cohort was consistent with this, with most patients having more than 12 times of IVI (69.231%). Very few patients refused postoperative IVI or received SI only (12.040%). The distribution of baseline data in the external validation cohort was similar to that in the training cohort. The baseline demographics of the study cohort are summarized in **Table 1**.

**Table 1:** Baseline demographics & clinical characteristics of the study population.

Variables	Level	Number of patients (%)	
		Training cohort	External validation cohort
*Gender*	Female	49 (16.388)	13 (11.111)
	Male	250 (83.612)	104 (88.889)
*Age (years)*	≤70	181 (60.535)	76 (64.957)
	>70	118 (39.465)	41 (35.043)
*BMI (kg/m^2^)*	<25	161 (53.846)	86 (73.504)
	≥25	138 (46.154)	31 (26.496)
*Smoking history before TURBT*	No	177 (59.197)	84 (71.795)
	Yes	122 (40.803)	33 (28.205)
*T category*	Ta	181 (60.535)	67 (57.265)
	T1	118 (39.465)	50 (42.735)
*Prior recurrence status*	Primary	264 (88.294)	95 (81.197)
	Recurrent	35 (11.706)	22 (18.803)
*Pathology grade*	Low	110 (36.789)	63 (53.846)
	High	189 (63.211)	54 (46.154)
*Tumor number*	Single	146 (48.829)	40 (34.188)
	Multiple	153 (51.171)	77 (65.812)
*Maximum tumor diameter (cm)*	<3	221 (73.913)	80 (68.376)
	≥3	78 (26.087)	37 (31.624)
*Gross hematuria*	No	60 (20.067)	24 (20.513)
	Yes	239 (79.933)	93 (79.487)
*Urine RBC*	0-25	104 (34.783)	41 (35.043)
	25-9000	159 (53.177)	62 (52.991)
	>9000	36 (12.040)	14 (11.966)
*Times of IVI*	0-1	36 (12.040)	14 (11.966)
	2-12	56 (18.729)	41 (35.043)
	>12	207 (69.231)	62 (52.991)
*NLR*	<3.18	253 (84.615)	97 (82.906)
	≥3.18	46 (15.385)	20 (17.094)
*PLR*	<179.29	263 (87.960)	99 (84.615)
	≥179.29	36 (12.040)	18 (15.385)
*LMR*	<2.88	43 (14.381)	34 (29.060)
	≥2.88	256 (85.619)	83 (70.940)
*dNLR*	<2.36	259 (86.622)	100 (85.470)
	≥2.36	40 (13.378)	17 (14.530)
*SII*	<525.26	215 (71.906)	60 (51.282)
	≥525.26	84 (28.094)	57 (48.718)
*PNI*	<52	141 (47.157)	79 (67.521)
	≥52	158 (52.843)	38 (32.479)
*De Ritis ratio*	<0.58	41 (13.712)	24 (20.513)
	≥0.58	258 (86.288)	93 (79.487)

BMI=body mass index=weight/height^2^; TURBT=transurethral resection of the bladder tumor; U-RBC=urine red blood cell count; IVI=intravesical instillation; NLR=neutrophil-lymphocyte ratio; PLR=platelet-lymphocyte ratio; LMR=lymphocyte-monocyte ratio; dNLR=derived neutrophil-lymphocyte ratio=neutrophil/(white blood cell-neutrophil); SII=systemic immune-inflammation index=platelet* neutrophil/lymphocyte; PNI=prognostic nutritional index=albumin+5* lymphocyte; De Ritis ratio=aspartate transaminase/alanine transaminase.

### The overlapping relationships and predictive values of tumor invasion locations

**Figure 2:**
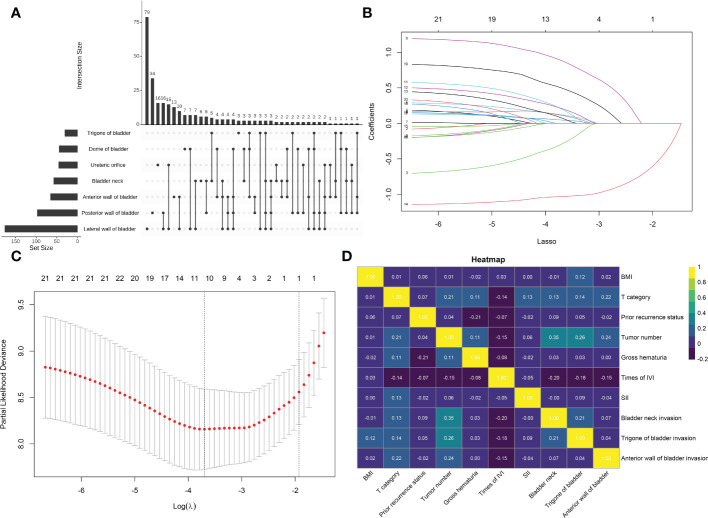
**(A)** An UpSet plot showing the intersections between tumor invasion locations. **(B)** Plot showing the ten-fold cross-validation *via* minimum criteria for the selection of the optimal value of tuning parameter (λ). Dotted vertical lines were drawn at the value with the minimum criteria and one standard error of the minimum criteria. **(C)** The least absolute shrinkage and selection operator coefficient profiles of the 21 clinicopathologic features associated with recurrence-free survival. A dotted vertical line was drawn at the optimal λ value identified through ten-fold cross-validation. The resulting 10 predictors with non-zero coefficients were identified based on the log (λ1se) value. **(D)** Heat map showing the correlation between the patients’ clinicopathologic features based on Spearman’s rank correlation coefficient. BMI, body mass index; IVI, intravesical instillation; SII, systemic immune-inflammation index = platelet* neutrophil/lymphocyte.

The overlapping relationships of NMIBC invasion locations are shown in **Figure 2A**. Most tumors invade only the lateral wall of the bladder (26.421%). At the same time, nearly 60% of the tumors occurred with lateral bladder wall invasion. The five most frequent tumor invasion locations were: lateral wall of the bladder only (26.421%), posterior wall of the bladder only (11.371%), ureteric orifice only (5.351%), lateral wall and posterior wall (5.351%), and lateral wall and ureteric orifice (5.016%). The odds of tumor involvement at these locations in descending order were: lateral wall of the bladder, posterior wall of the bladder, anterior wall of the bladder, bladder neck, ureteric orifice, dome of the bladder, and trigone of the bladder. All three invasion locations that were statistically significant in the univariate regression analysis (bladder neck, trigone of the bladder, and anterior wall of the bladder) were retained after the LASSO regression (**Table 2**).

**Table 2:** Baseline characteristics and univariable Cox regression analysis of tumor invasion locations.

Variables	N (%)	HR	95% CI	p
*Bladder neck*				**<0.001**
*No invasion*	242 (80.936)	1(reference)		
*Invasion*	57 (19.064)	2.699	[1.598,4.558]	
*Trigone of bladder*				**0.01**
*No invasion*	269 (89.967)	1(reference)		
*Invasion*	30 (10.033)	2.394	[1.230,4.661]	
*Dome of bladder*				0.855
*No invasion*	255 (85.284)	1(reference)		
*Invasion*	44 (14.716)	1.063	[0.551,2.051]	
*Lateral wall of bladder*				0.085
*No invasion*	123 (41.137)	1(reference)		
*Invasion*	176 (58.863)	0.646	[0.392,1.063]	
*Anterior wall of bladder*				**0.01**
*No invasion*	234 (78.261)	1(reference)		
*Invasion*	65 (21.739)	1.991	[1.180,3.360]	
*Posterior wall of bladder*				0.526
*No invasion*	202 (67.559)	1(reference)		
*Invasion*	97 (32.441)	1.187	[0.699,2.015]	
*Ureteri corifice*				0.555
*No invasion*	254 (84.950)	1(reference)		
*Invasion*	45 (15.050)	0.799	[0.380,1.682]	

N=number of patients; HR, hazard ratio; CI, confidence interval. Bolded p-values indicate statistically significant correlations (p < 0.05).

### Screening for predictive factors

**Figure 3:**
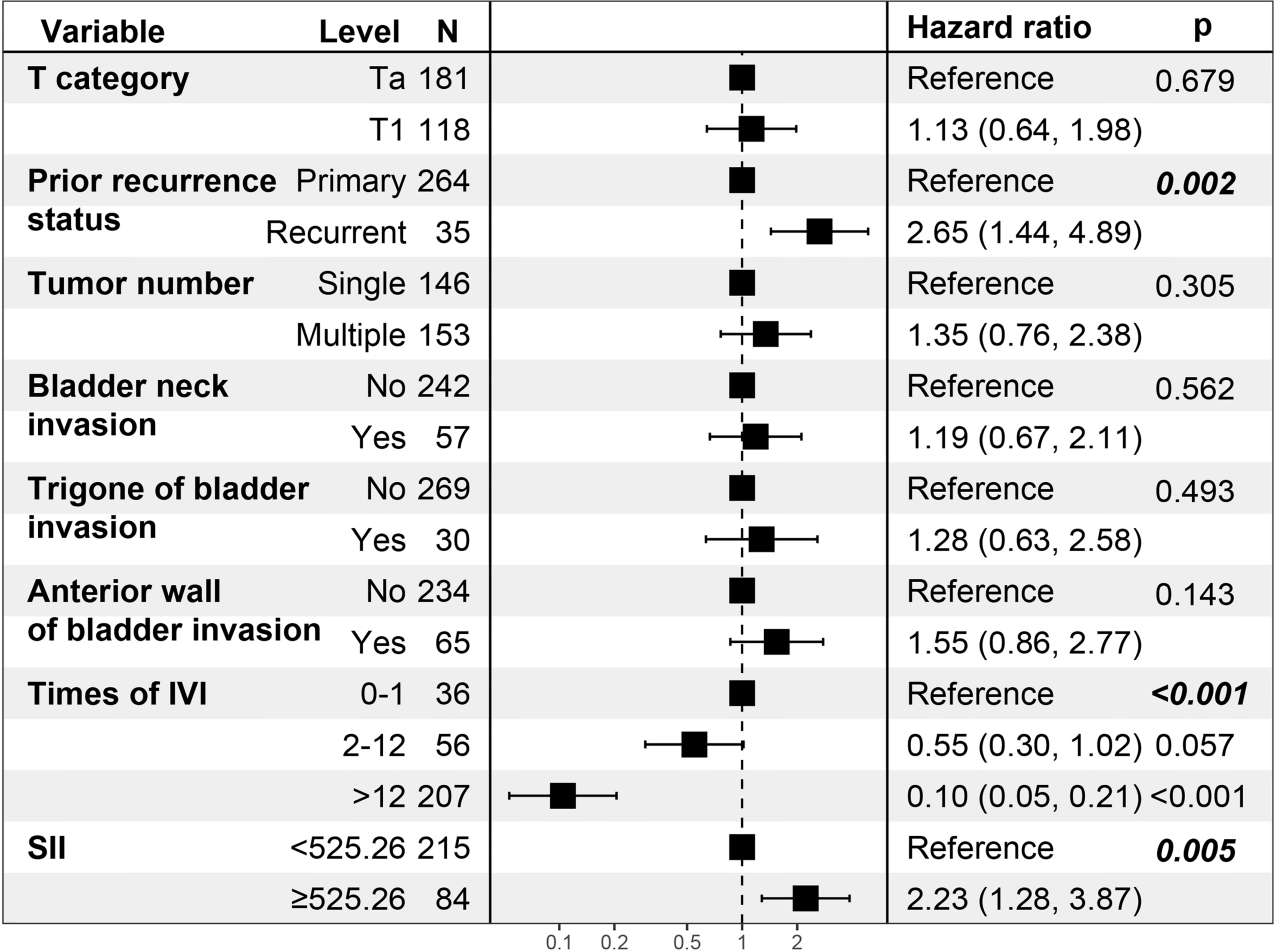
Multivariate Cox regression analysis of the patients’ clinicopathologic features. N, number of patients; IVI, intravesical instillation; SII, systemic immune-inflammation index = platelet* neutrophil/lymphocyte.

By including 21 clinicopathologic features in the LASSO regression analysis, 10 candidates with non-zero coefficients that are associated with RFS were identified (**Figures 2B, C**). The correlations between these variables were analyzed and visualized by a heat map using Spearman’s rank correlation coefficient (**Figure 2D**), which showed no significant correlations between these variables. These potential predictors were then included in the univariate Cox regression analyses. In the univariate regression (**Tables 2**, **3**), T category, prior recurrence status, tumor number, times of IVI, SII, bladder neck invasion, trigone of the bladder invasion, and anterior wall of the bladder invasion were suggested to be associated with RFS (all p<0.05). The statistically significant variables in the univariate analysis were then assessed using multivariate Cox regression, which showed that prior recurrence status (HR [95% CI]: 2.65 [1.44-4.89], p=0.002), times of IVI (p<0.001), and SII (HR [95% CI]: 2.23 [1.28-3.87], p=0.005) were independent predictors of RFS (**Figure 3**).

**Table 3:** Univariable Cox regression analysis after the LASSO regression analysis, without tumor invasion locations.

Variables	N	HR	95% CI	p
*BMI*				0.214
*<25*	161			
*≥25*	138	0.723	[0.434,1.206]	
*T category*				**0.002**
*Ta*	181	1 (reference)		
*T1*	118	2.206	[1.332,3.652]	
*Prior recurrence status*				**<0.001**
*Primary*	264	1 (reference)		
*Recurrent*	35	3.369	[1.941,5.847]	
*Tumor number*				**0.016**
*Single*	146	1 (reference)		
*Multiple*	153	1.911	[1.127,3.241]	
*Gross hematuria*				0.171
*No*	60	1 (reference)		
*Yes*	239	1.682	[0.800,3.538]	
*Times of IVI*				**<0.001**
*0-1*	36	1 (reference)		
*2-12*	56	0.534	[0.296,0.961]	0.036
*>12*	207	0.081	[0.043,0.152]	<0.001
*SII*				**0.003**
*<525.26*	215	1(reference)		
*≥525.26*	84	2.17	[1.308,3.599]	

HR, hazard ratio; CI, confidence interval; BMI, body mass index, weight/height^2^; IVI,intravesical instillation; PLR, platelet-lymphocyte ratio; SII, systemic immune-inflammation index,platelet* neutrophil/lymphocyte; De Ritis ratio, aspartate transaminase/alanine transaminase. Bolded p-values indicate statistically significant correlations (p < 0.05).

### Development of the nomogram

**Figure 4:**
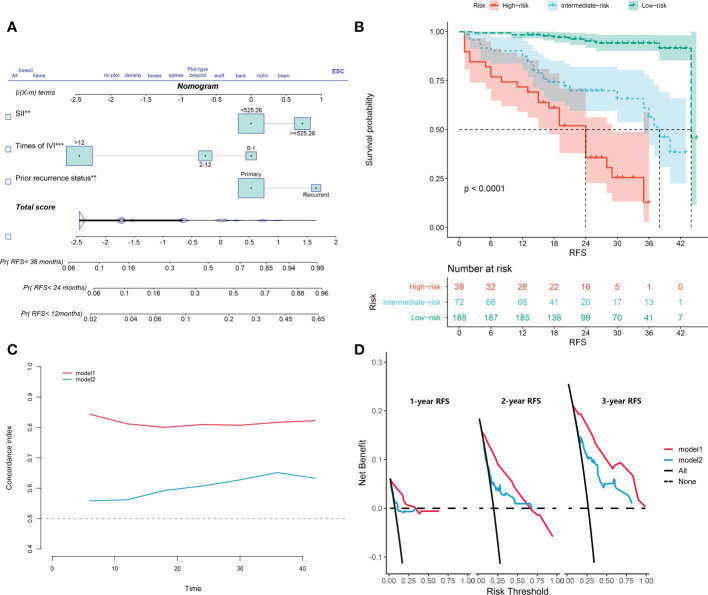
**(A)** The constructed nomogram for predicting recurrence-free survival of non-muscle-invasive bladder cancer patients after transurethral resection of the bladder tumor. **(B)** Kaplan-Meier curves of low-risk, intermediate-risk, and high-risk groups based on the prediction of the nomogram. **(C)** Harrell’s concordance index for 1-, 2-, and 3-year recurrence-free survival of two models. **(D)** Decision-curve analyses demonstrate the net benefit of using the models. Model 1, the nomogram; model 2, model based on age, gender, T category, prior recurrence status, pathology grade, tumor number, and maximum tumor diameter. IVI, intravesical instillation; SII, systemic immune-inflammation index = platelet* neutrophil/lymphocyte.

The nomogram was constructed based on the three identified variables (**Figure 4A**). Based on this model, all included subjects were scored individually. Patients were then classified as low-risk (<29.82 points), intermediate-risk (29.82-100.00 points), or high-risk (>100.00 points). The Kaplan–Meier survival analysis showed that the patients in the high-risk group had significant worse RFS than patients in the other two groups (p<0.0001; **Figure 4B**).

### Validation of the nomogram

**Figure 5:**
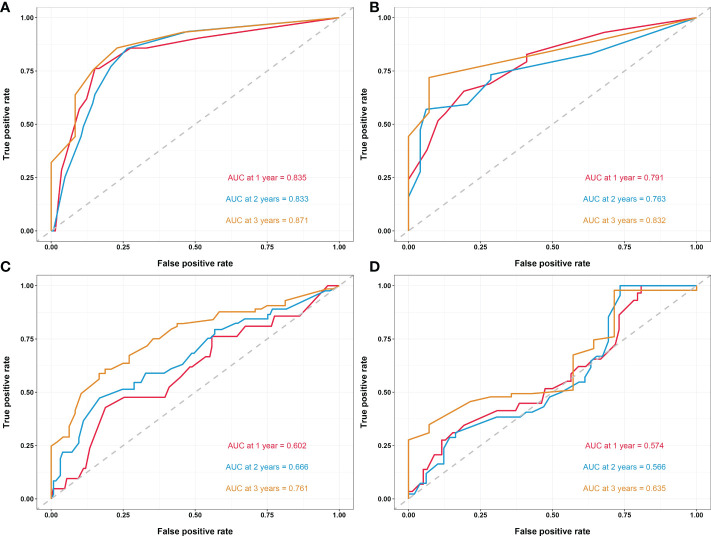
**(A)** Time-dependent ROC curves of the nomogram in the training cohort. **(B)** Time-dependent ROC curves of the nomogram in the external validation cohort. **(C)** Time-dependent ROC curves of model 2 in the training cohort. **(D)** Time-dependent ROC curves of model 2 in the external validation cohort. Model 2, model based on age, gender, T category, prior recurrence status, pathology grade, tumor number, and maximum tumor diameter. ROC, receiver operating characteristic; AUC, area under the curve.

**Figure 6:**
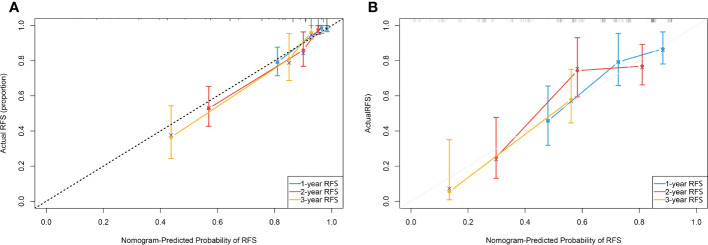
**(A)** Calibration plot of the nomogram done by bootstrapping with 1,000 resamples for predicting recurrence-free survival(RFS) in the training cohort. **(B)** Calibration plot of the nomogram done by bootstrapping with 1,000 resamples for predicting recurrence-free survival in the external validation cohort.

In another model, we incorporated the variables included in the EORTC and CUETO systems (age at diagnosis, gender, T category, number of tumors, tumor size, pathological grade, and previous recurrence status). We defined it as model 2 and compared it to our nomogram model (model 1). The IDI (16%) indicated a significant improvement in the performance of model 1 compared to model 2. The C-index curve at different time points of model 1 was significantly higher than model 2 (**Figure 4C**). The DCA curve demonstrated that the net benefit associated with the utilization of model 1 is better than in model 2 (**Figure 4D**). The ROC curves showed that model 1 had an excellent predictive accuracy of the 1-, 2-, and 3-year RFS rates, with AUC reaching 0.835, 0.833, and 0.871, respectively (**Figure 5A**). The AUC of model 1 in the external validation cohort also showed great performance (**Figure 5B**). Moreover, model 1 is significantly more effective than model 2 in both the training and the external validation cohorts (**Figures 5C, D**). The calibration plots validated by 1,000 bootstrap resampling also proved the appreciable reliability of model 1 in both internal and external validation cohorts (**Figure 6**).

## Discussion

Our study investigated the overlapping relationships of tumor invasion locations. Furthermore, for the first time, U-RBC and times of IVI were included in the predictive assessment, and a model was established to predict RFS in NMIBC patients after TURBT by combining the patient’s clinicopathological features and hematological indicators.

SI has been proven to be critical in reducing the recurrence rate of NMIBC by destroying circulating tumor cells after TURBT and removing residual tumor cells at the surgical location and microscopic tumors that were not detected intraoperatively (38, 39). However, the duration and frequency of IVI after TURBT have not been conclusively established (13). For the first time, in this study, we collected the total times patients received IVI and divided them into three groups. The results showed that patients with >12 times of IVI had a significantly better prognosis than those who refused postoperative IVI or received SI only (HR [95% CI]: 0.10 [0.05-0.21], p<0.001). Our findings suggest that it is necessary to receive regular and sufficient IVI after TURBT, especially for high-risk patients.

The inflammatory state in cancer patients is thought to be one of the main causes of the proliferative properties of tumor cells. This state promotes the supply of multiple bioactive substances, including growth factors, chemokines, and cytokines, to the tumor microenvironment. Thus promoting the development of pathological states such as immune escape, immune metastasis and angiogenesis (40). SIR, which is usually measured by surrogate blood parameters, has been shown to independently predict survival outcomes in patients with various malignancies (41–43). Ohno et al. gave an overview of the role of SIR indicators such as NLR and PNI in the prognosis of NMIBC and suggested that abnormal fluctuations in preoperative SIR levels may be closely associated with patient survival (20). Furthermore, Tang et al. included additional SIR indicators and showed that elevated NLR, PLR, SII, and reduced PNI, all led to higher pathological grade and aggressiveness in NMIBC patients at the time of first diagnosis (21). Our results suggest that SII is an independent predictor of RFS in NMIBC patients. Specifically, patients with elevated SII (≥525.26) have a significantly increased risk of tumor recurrence or progression (HR [95% CI]: 2.23 [1.28-3.87], p=0.005), which is consistent with published literature (27–30).

Most of the published literature only included subjects with a single invasion location and excluded patients with multiple lesion locations (13–16, 44–46). Furthermore, several studies suggested that patients with bladder neck invasion may have the worst prognosis (44–46). However, many studies found different conclusions. Svatek et al. studied patients undergoing radical cystectomy and showed that patients who invaded the bladder triangle had a greater risk of intraoperative lymph node metastasis and a worse cancer-specific survival rate (14). Meanwhile, Weiner et al. found that for patients with bladder cancer treated with chemoradiotherapy, the trigone of bladder invasion was associated with worse survival (15). Furthermore, Vukomanovic et al. found that tumors in the lateral and posterior bladder walls may have a higher risk of recurrence when treated with TURBT only (16). Whereas, the study by Martin et al. concluded that the invasion of the dome of the bladder was an independent risk factor for poor prognosis (17). On the other hand, Ahmadi et al. found that overall survival was worse in patients with an invasion of the posterior bladder wall (18). In this study, we have constructed one UpSet diagram to visualize the overlapping relationships between different tumor invasion conditions. The results indicated that the five most frequent tumor invasion conditions are the lateral wall of the bladder only, the posterior wall of the bladder only, the ureteric orifice only, the lateral wall and posterior wall of the bladder, and the lateral wall and ureteric orifice of the bladder, in order. The odds of tumor involvement at the location in descending order are lateral wall of the bladder, the posterior wall of the bladder, the anterior wall of the bladder, the bladder neck, the ureteric orifice, the dome of the bladder, and the trigone of the bladder. These findings are broadly consistent with previous statistics (13, 15). Univariate COX regression analysis showed that patients had significantly worse RFS when the tumor has invaded the bladder neck (HR [95% CI]:2.699 [1.598,4.558], p<0.001), trigone (HR [95% CI]:2.394 [1.230,4.661], p=0.01), or anterior wall (HR [95% CI]:1.991 [1.180,3.360], p=0.01). We propose, based on clinical experience, that tumors in the bladder neck, bladder triangle, or anterior bladder wall have restricted fields of view when TURBT is performed in the lithotomy position, which subsequently increases the risk of tumor residual. Also, the width of the inner bladder wall may be thinner in these areas, increasing the possibility of deep infiltration. Unfortunately, in the multivariate analysis, none of these three invasion locations was statistically significant. However, we believe this variable deserves further exploration in the future.

In 2019, Kim et al. first proposed gross hematuria as a valuable predictor of NMIBC recurrence (31). We included gross hematuria while also collecting U-RBC samples of patients after admission. To our knowledge, we are the first study to assess the value of U-RBC in the prognosis of patients with NMIBC. We found that gross hematuria was not statistically significant in the univariate regression analysis (HR [95% CI]: 1.682 [0.800,3.538], p=0.171). However, it is worth mentioning that U-RBC, despite being excluded from the LASSO regression analysis, was found to be a possible independent risk factor for predicting RFS in our univariate analysis (p=0.009). We will continue to discuss the value of this variable in the prognosis of NMIBC patients in our future studies. We hypothesize that elevated U-RBC in routine urinalysis might mean that the tumor has an extensive invasion of the microvasculature, which indicates the strong aggressiveness of the tumor.

The limitations of this study are inherent to its retrospective, observational design and associated biases. First of all, our sample size was not large enough due to some patients not having complete medical information and follow-ups. However, we tried to compensate for this through external validation in collaboration with other medical centers. Secondly, the identification of concurrent CIS is time-consuming, and we are working with pathologists to incorporate this variable in a follow-up study. Meanwhile, we did not include different IVI regimens in the study due to the inability of some patients to provide accurate perfusion drugs, especially due to the presence of patients who received BCG or mixed multiple chemotherapy drugs, which in turn may have led to potential heterogeneity between subjects. Furthermore, the exact times of the bladder induction perfusion and bladder persistence perfusion should be further confirmed. Lastly, the period of follow-up is too short, and the initial time of follow-up should be extended further.

In conclusion, in this study, we established a model based on patients’ prior recurrence status and times of IVI and SII to predict RFS in NMIBC patients after TURBT. We believe that this risk calculator based on the model can be applied as an ideal clinically personalized tool, which can offer reliable prognostic information and treatment strategies for patients in tiered management to obtain the maximum survival benefit.

## Data availability statement

The raw data supporting the conclusions of this article will be made available by the authors, without undue reservation.

## Ethics statement

The studies involving human participants were reviewed and approved by the Ethics Committee of the Affiliated Hospital of Xuzhou Medical University (XYFT2022-KL340-01) and the Ethics Committee of the First Affiliated Hospital of Guangxi Medical University (2022-E318-01). Written informed consent for participation was not required for this study in accordance with the national legislation and the institutional requirements.

## Author contributions

LD and JW contributed to the idea and design. XD, WX, and KW followed up the patients. YangZ, YanZ, and XS collected and organized the data. LD analyzed the data and drew the figures and tables. LD and JW wrote the draft and contributed to manuscript writing and revision. All authors approved the final manuscript as submitted.

## Funding

This study was funded by the second round of Xuzhou Medical Leading Talents Training Project (XWRCHT20210027).

## Acknowledgments

We thank Bullet Edits Limited for the linguistic editing and proofreading of the manuscript.

## Conflict of interest

The authors declare that the research was conducted in the absence of any commercial or financial relationships that could be construed as a potential conflict of interest.

## Publisher’s note

All claims expressed in this article are solely those of the authors and do not necessarily represent those of their affiliated organizations, or those of the publisher, the editors and the reviewers. Any product that may be evaluated in this article, or claim that may be made by its manufacturer, is not guaranteed or endorsed by the publisher.

## References

[1] Siegel RL Miller KD Fuchs HE Jemal A . Cancer statistics, 2022. CA Cancer J Clin (2022) 72:7–33. doi: 10.3322/caac.21708 35020204

[2] Babjuk M Burger M Capoun O Cohen D Compérat EM Dominguez Escrig JL . European Association of urology guidelines on non–muscle-invasive bladder cancer (ta, t1, and carcinoma in situ). Eur Urol (2022) 81:75–94. doi: 10.1016/j.eururo.2021.08.010 34511303

[3] Sylvester RJ van der Meijden APM Oosterlinck W Witjes JA Bouffioux C Denis L . Predicting recurrence and progression in individual patients with stage ta t1 bladder cancer using eortc risk tables: a combined analysis of 2596 patients from seven eortc trials. Eur Urol (2006) 49:466–77. doi: 10.1016/j.eururo.2005.12.031 16442208

[4] Fernandez-Gomez J Madero R Solsona E Unda M Martinez-Piñeiro L Gonzalez M . Predicting nonmuscle invasive bladder cancer recurrence and progression in patients treated with bacillus calmette-guerin: the cueto scoring model. J Urol (2009) 182:2195–203. doi: 10.1016/j.juro.2009.07.016 19758621

[5] Fujii Y . Prediction models for progression of non-muscle-invasive bladder cancer: a review. Int J Urol (2018) 25:212–8. doi: 10.1111/iju.13509 29247553

[6] Bree KK Shan Y Hensley PJ Lobo N Hu C Tyler DS . Management, surveillance patterns, and costs associated with low-grade papillary stage ta non–muscle-invasive bladder cancer among older adults, 2004-2013. JAMA Netw Open (2022) 5:e223050. doi: 10.1001/jamanetworkopen.2022.3050 35302627 PMC8933744

[7] Koie T Ohyama C Hosogoe S Yamamoto H Imai A Hatakeyama S . Oncological outcomes of a single but extensive transurethral resection followed by appropriate intra-vesical instillation therapy for newly diagnosed non-muscle-invasive bladder cancer. Int Urol Nephrol (2015) 47:1509–14. doi: 10.1007/s11255-015-1048-3 26149637

[8] Brausi M Witjes JA Lamm D Persad R Palou J Colombel M . A review of current guidelines and best practice recommendations for the management of nonmuscle invasive bladder cancer by the international bladder cancer group. J Urol (2011) 186:2158–67. doi: 10.1016/j.juro.2011.07.076 22014799

[9] Konety B Isharwal S . Non-muscle invasive bladder cancer risk stratification. Indian J Urol (2015) 31:289. doi: 10.4103/0970-1591.166445 26604439 PMC4626912

[10] Burger M Catto JWF Dalbagni G Grossman HB Herr H Karakiewicz P . Epidemiology and risk factors of urothelial bladder cancer. Eur Urol (2013) 63:234–41. doi: 10.1016/j.eururo.2012.07.033 22877502

[11] Teoh JY Huang J Ko WY Lok V Choi P Ng C . Global trends of bladder cancer incidence and mortality, and their associations with tobacco use and gross domestic product per capita. Eur Urol (2020) 78:893–906. doi: 10.1016/j.eururo.2020.09.006 32972792

[12] Laaksonen MA MacInnis RJ Canfell K Giles GG Hull P Shaw JE . The future burden of kidney and bladder cancers preventable by behavior modification in australia: a pooled cohort study. Int J Cancer (2019) 146:874–83. doi: 10.1002/ijc.32420 31107541

[13] Stephenson WT Holmes FF Noble MJ Gerald KB . Analysis of bladder carcinoma by subsite. cystoscopic location may have prognostic value. Cancer (1990) 66:1630–5. doi: 10.1002/1097-0142(19901001)66:7<1630::aid-cncr2820660730>3.0.co;2-7 2208014

[14] Svatek RS Clinton TN Wilson CA Kamat AM Grossman HB Dinney CP . Intravesical tumor involvement of the trigone is associated with nodal metastasis in patients undergoing radical cystectomy. Urology (2014) 84:1147–51. doi: 10.1016/j.urology.2014.05.011 25174656

[15] Weiner AB Desai AS Meeks JJ . Tumor location may predict adverse pathology and survival following definitive treatment for bladder cancer: a national cohort study. Eur Urol Oncol (2019) 2:304–10. doi: 10.1016/j.euo.2018.08.018 31200845

[16] Vukomanovic I Colovic V Soldatovic I Hadzi-Djokic J . Prognostic significance of tumor location in high-grade non-muscle-invasive bladder cancer. Med Oncol (2012) 29:1916–20. doi: 10.1007/s12032-011-9999-4 21656270

[17] Martin M Bernardini S Kleinclauss F Della NE Henry PC Bittard H . Prognostic value of tumor location of urothelial tumors of the bladder, after total cystectomy. Prog Urol (2002) 12:1221–7. 12545628

[18] Ahmadi H Miranda G Cai J Daneshmand S . Intravesical location of the tumor: How does it affect the pattern of lymph node metastasis and oncological outcome in urothelial cancer of bladder? J Urol (2013) 1769 189:e727. doi: 10.1016/j.juro.2013.02.2898 23594643

[19] Cantiello F Russo GI Vartolomei MD Farhan ARA Terracciano D Musi G . Systemic inflammatory markers and oncologic outcomes in patients with high-risk non–muscle-invasive urothelial bladder cancer. Eur Urol Oncol (2018) 1:403–10. doi: 10.1016/j.euo.2018.06.006 31158079

[20] Ohno Y . Role of systemic inflammatory response markers in urological malignancy. Int J Urol (2019) 26:31–47. doi: 10.1111/iju.13801 30253448

[21] Tang X Cao Y Liu J Wang S Yang Y Du P . Diagnostic value of inflammatory factors in pathology of bladder cancer patients. Front Mol Biosci (2020) 7:575483. doi: 10.3389/fmolb.2020.575483 33251247 PMC7674661

[22] Yıldız HA Değer MD Aslan G . Prognostic value of preoperative inflammation markers in non-muscle invasive bladder cancer. Int J Clin Pract (2021) 75:e14118. doi: 10.1111/ijcp.14118 33636055

[23] Mano R Baniel J Shoshany O Margel D Bar-On T Nativ O . Neutrophil-to-lymphocyte ratio predicts progression and recurrence of non–muscle-invasive bladder cancer. Urologic Oncology: Semin Original Investigations (2015) 33:61–7. doi: 10.1016/j.urolonc.2014.06.010 25060672

[24] Mbeutcha A Shariat SF Rieken M Rink M Xylinas E Seitz C . Prognostic significance of markers of systemic inflammatory response in patients with non–muscle-invasive bladder cancer. Urologic Oncology: Semin Original Investigations (2016) 34:417–83. doi: 10.1016/j.urolonc.2016.05.013 27646875

[25] Ogihara K Kikuchi E Yuge K Yanai Y Matsumoto K Miyajima A . The preoperative neutrophil-to-lymphocyte ratio is a novel biomarker for predicting worse clinical outcomes in non-muscle invasive bladder cancer patients with a previous history of smoking. Ann Surg Oncol (2016) 23:1039–47. doi: 10.1245/s10434-016-5578-4 27660257

[26] Vartolomei MD Porav-Hodade D Ferro M Mathieu R Abufaraj M Foerster B . Prognostic role of pretreatment neutrophil-to-lymphocyte ratio (nlr) in patients with non–muscle-invasive bladder cancer (nmibc): a systematic review and meta-analysis. Urologic Oncology: Semin Original Investigations (2018) 36:389–99. doi: 10.1016/j.urolonc.2018.05.014 29884342

[27] Katayama S Mori K Pradere B Laukhtina E Schuettfort VM Quhal F . Prognostic value of the systemic immune-inflammation index in non-muscle invasive bladder cancer. World J Urol (2021) 39:4355–61. doi: 10.1007/s00345-021-03740-3 PMC8602174 34143284

[28] Ke Z Chen H Chen J Cai H Lin Y Sun X . Preoperative abdominal fat distribution and systemic immune inflammation were associated with response to intravesical bacillus calmette-guerin immunotherapy in patients with non-muscle invasive bladder cancer. Clin Nutr (2021) 40:5792–801. doi: 10.1016/j.clnu.2021.10.019 34775222

[29] Bi H Shang Z Jia C Wu J Cui B Wang Q . Predictive values of preoperative prognostic nutritional index and systemic immune-inflammation index for long-term survival in high-risk non-muscle-invasive bladder cancer patients: a single-centre retrospective study. Cancer Manag Res (2020) 12:9471–83. doi: 10.2147/CMAR.S259117 PMC7534864 33061634

[30] Zhao R Shan J Nie L Yang X Yuan Z Xu H . The predictive value of the ratio of the product of neutrophils and hemoglobin to lymphocytes in non-muscular invasive bladder cancer patients with postoperative recurrence. J Clin Lab Anal (2021) 35:e23883. doi: 10.1002/jcla.23883 34184796 PMC8373351

[31] Kim HS Jeong CW Kwak C Kim HH Ku JH . Novel nomograms to predict recurrence and progression in primary non-muscle-invasive bladder cancer: validation of predictive efficacy in comparison with european organization of research and treatment of cancer scoring system. World J Urol (2019) 37:1867–77. doi: 10.1007/s00345-018-2581-3 30535715

[32] Kluth LA Xylinas E Crivelli JJ Passoni N Comploj E Pycha A . Obesity is associated with worse outcomes in patients with t1 high grade urothelial carcinoma of the bladder. J Urol (2013) 190:480–6. doi: 10.1016/j.juro.2013.01.089 23376707

[33] Ferro M Vartolomei MD Russo GI Cantiello F Farhan ARA Terracciano D . An increased body mass index is associated with a worse prognosis in patients administered bcg immunotherapy for t1 bladder cancer. World J Urol (2019) 37:507–14. doi: 10.1007/s00345-018-2397-1 29992381

[34] Roupret M Babjuk M Burger M Capoun O Cohen D Comperat EM . European Association of urology guidelines on upper urinary tract urothelial carcinoma: 2020 update. Eur Urol (2021) 79:62–79. doi: 10.1016/j.eururo.2020.05.042 32593530

[35] Pencina MJ D'Agostino RS D'Agostino RJ Vasan RS . Evaluating the added predictive ability of a new marker: from area under the roc curve to reclassification and beyond. Stat Med (2008) 27:157–72, 207-12. doi: 10.1002/sim.2929 17569110

[36] Van Calster B Wynants L Verbeek J Verbakel JY Christodoulou E Vickers AJ . Reporting and interpreting decision curve analysis: a guide for investigators. Eur Urol (2018) 74:796–804. doi: 10.1016/j.eururo.2018.08.038 30241973 PMC6261531

[37] Sylvester RJ Rodríguez O Hernández V Turturica D Bauerová L Bruins HM . European Association of urology (eau) prognostic factor risk groups for non–muscle-invasive bladder cancer (nmibc) incorporating the who 2004/2016 and who 1973 classification systems for grade: an update from the eau nmibc guidelines panel. Eur Urol (2021) 79:480–8. doi: 10.1016/j.eururo.2020.12.033 33419683

[38] Brocks CP Büttner H Böhle A . Inhibition of tumor implantation by intravesical gemcitabine in a murine model of superficial bladder cancer. J Urol (2005) 174:1115–8. doi: 10.1097/01.ju.0000168657.51551.49 16094076

[39] Oosterlinck W Kurth KH Schroder F Bultinck J Hammond B Sylvester R . A prospective european organization for research and treatment of cancer genitourinary group randomized trial comparing transurethral resection followed by a single intravesical instillation of epirubicin or water in single stage ta, t1 papillary carcinoma of the bladder. J Urol (1993) 149:749–52. doi: 10.1016/s0022-5347(17)36198-0 8455236

[40] Hanahan D Weinberg RA . Hallmarks of cancer: the next generation. Cell (2011) 144:646–74. doi: 10.1016/j.cell.2011.02.013 21376230

[41] Roxburgh CS McMillan DC . Role of systemic inflammatory response in predicting survival in patients with primary operable cancer. Future Oncol (2010) 6:149–63. doi: 10.2217/fon.09.136 20021215

[42] Rajwa P Zyczkowski M Paradysz A Slabon-Turska M Suliga K Bujak K . Novel hematological biomarkers predict survival in renal cell carcinoma patients treated with nephrectomy. Arch Med Sci (2020) 16:1062–71. doi: 10.5114/aoms.2017.70250 PMC7444725 32863995

[43] Attawettayanon W Choorit T Chalieopanyarwong V Pripatnanont C . Significance of preoperative hematologic scoring in predicting death among patients with non-metastatic renal cell carcinoma undergoing nephrectomy. Asian J Surg (2021) 44:952–6. doi: 10.1016/j.asjsur.2021.01.029 33622600

[44] Kobayashi S Fujii Y Koga F Yokoyama M Ishioka J Matsuoka Y . Impact of bladder neck involvement on progression in patients with primary non–muscle invasive bladder cancer: a prospective validation study. Urologic Oncology: Semin Original Investigations (2014) 32:29–38. doi: 10.1016/j.urolonc.2013.04.001 23702087

[45] Zhan X Guo J Chen L Deng W Liu X Zhu K . Prognostic significance of bladder neck involvement in non-muscle-invasive bladder cancer: a seer database analysis with 19,919 patients. Cancer Med (2021) 10:6868–80. doi: 10.1002/cam4.4219 PMC8495274 34423585

[46] Fukushima H Moriyama S Waseda Y Fukuda S Uehara S Tanaka H . Significance of bladder neck involvement in risk substratification of intermediate-risk non–muscle-invasive bladder cancer. Eur Urol Focus (2021) 7:366–72. doi: 10.1016/j.euf.2020.01.006 31987764

